# Implementing value-based healthcare using a digital health exchange platform to improve pregnancy and childbirth outcomes in urban and rural Kenya

**DOI:** 10.3389/fpubh.2022.1040094

**Published:** 2022-11-17

**Authors:** Peter Dohmen, Teresa De Sanctis, Emma Waiyaiya, Wendy Janssens, Tobias Rinke de Wit, Nicole Spieker, Mark Van der Graaf, Erik M. Van Raaij

**Affiliations:** ^1^Rotterdam School of Management, Erasmus University Rotterdam, Rotterdam, Netherlands; ^2^Erasmus School of Health Policy & Management, Erasmus University Rotterdam, Rotterdam, Netherlands; ^3^PharmAccess Foundation, Amsterdam, Netherlands; ^4^PharmAccess Foundation, Nairobi, Kenya; ^5^School of Business and Economics, VU Amsterdam, Amsterdam, Netherlands; ^6^Amsterdam Institute for Global Health and Development, University of Amsterdam, Amsterdam, Netherlands

**Keywords:** value-based healthcare, MNCH, cohort-based implementation, digital health, outcome measurement, LMIC

## Abstract

Maternal and neonatal mortality rates in many low- and middle-income countries (LMICs) are still far above the targets of the United Nations Sustainable Development Goal 3. Value-based healthcare (VBHC) has the potential to outperform traditional supply-driven approaches in changing this dismal situation, and significantly improve maternal, neonatal and child health (MNCH) outcomes. We developed a theory of change and used a cohort-based implementation approach to create short and long learning cycles along which different components of the VBHC framework were introduced and evaluated in Kenya. At the core of the approach was a value-based care bundle for maternity care, with predefined cost and quality of care using WHO guidelines and adjusted to the risk profile of the pregnancy. The care bundle was implemented using a digital exchange platform that connects pregnant women, clinics and payers. The platform manages financial transactions, enables bi-directional communication with pregnant women *via* SMS, collects data from clinics and shares enriched information *via* dashboards with payers and clinics. While the evaluation of health outcomes is ongoing, first results show improved adherence to evidence-based care pathways at a predictable cost per enrolled person. This community case study shows that implementation of the VBHC framework in an LMIC setting is possible for MNCH. The incremental, cohort-based approach enabled iterative learning processes. This can support the restructuring of health systems in low resource settings from an output-driven model to a value based financing-driven model.

## Introduction

Over the past decade, investments in improving maternal, neonatal and child health (MNCH) in low- and middle-income countries (LMICs) have largely focused on reducing financial and geographical barriers to access skilled delivery and incentivizing adherence to antenatal guidelines improving scale, scope and quality of healthcare delivery and stimulating utilization by reducing financial barriers (1, 2). Despite these investments, maternal mortality rates (MMR) and neonatal mortality rates (NMR) are still high in LMICs. The latest figures show an MMR of 253 per 100,000 live births (3) and an NMR of 22 per 1,000 live births (4) in LMICs, while the United Nations Sustainable Development Goal 3 (SDG 3) targets specify an MMR of 70 (target 3.1) and an NMR of 12 (target 3.2) in 2030. This raises the question what more is required to meet patients' needs and to achieve the targets of SDG 3? Research shows that improving accessibility and adherence alone does not guarantee better outcomes and that a broader set of interventions aimed at quality of care is needed (2, 5, 6).

Transforming health systems toward a quality health system is a complex and long-term process that requires a multifaceted approach (7–9). Kruk et al. (1) argues that to achieve better outcomes in LMICs, health systems are needed that focus on patient-centeredness, resilience, equity and efficiency. A complicating factor is that in most LMICs, the organization and financing of healthcare is supply driven (10). To create high-quality health systems, scholars and practitioners argue that health systems should transition from a supply-driven model toward a value-driven model (11–13). In high-income countries (HICs) this approach is gaining momentum and some countries are implementing delivery models that embrace a value-driven approach (14, 15). However, in LMICs, value-driven service delivery models are not common and experiments with value-driven models are scant (16). This is remarkable as financial resources are limited in LMICs, and models that incentivize high-quality care at lower costs could be an answer to the question “how to do more with less” to improve MNCH outcomes.

Research on implementation and evaluation of MNCH service models in LMICs focusses mainly on output-based interventions, addressing single components or subsets of the VBHC framework, such as outcome measurements (16–18), performance-based payments (19, 20), (data-driven) quality improvements (21–23) or redesigns of referral systems (24, 25). In this paper we describe the development and implementation process of a value-based healthcare (VBHC) based digital MNCH care bundle called MomCare, developed by the international non-governmental organization (NGO) PharmAccess Foundation (PAF) specifically designed for LMICs.

The conceptual framework of MomCare is based on the concept of VBHC introduced by Porter and Teisberg (11). Value is defined as outcomes that matter to patients relative to the total costs of care delivery (26). In VBHC there is a strong focus on comprehensive outcome measurements (both clinical outcomes as well as patient-reported outcomes) and reimbursement systems that incentivize providers to maximize value (27). Importantly, value is created at the level of medical conditions or specific subpopulations, over full care cycles and providers should structure their organizations around patients' needs (26). VBHC is becoming a trend in transforming health systems in HICs as the first results of VBHC implementations seem to be positive (28–32). However, there are methodological and operational challenges to overcome when implementing VBHC in other settings. For example, the VBHC framework does not provide an implementation methodology (33, 34), patient-reported outcome measurements (PROMs) are sensitive to cultural variation and context-specific conditions (35), VBHC requires a cultural change within organizations with physicians becoming accountable for the full cycle of care (36) and a successful implementation requires leadership, clinical and managerial support, as well as substantial investments to enable data collection and analysis (16, 35).

## Context

In Kenya, 1.4 million babies were born in 2019 (37). The MMR is 342 and the NMR is 21 (4). Of pregnant women, 96% attended at least one antenatal care visit, and 58% attended antenatal care at least four times while 62% attended a skilled delivery (38). However, MNCH services remain highly inequitable (39). Healthcare services are provided by six levels of facilities, ranging from community services (level 1) to national referral hospitals and large private teaching hospitals (level 6). Antenatal and postnatal care services, including immunizations, are provided by most level 2–6 facilities such as dispensaries, maternity clinics and hospitals. Delivery services, including cesarean sections (C-sections), are mainly provided by maternity health centers (level 3), (sub)county referral hospitals and medium and large-sized private hospitals (levels 4 and 5) (40).

The penetration of mobile phones in Kenya is among the highest in African countries. As many as 97% of adults report to own or share a mobile phone (41). A large share (40%) of these however own a basic phone which cannot connect to the internet (41).

## The MomCare program

MomCare started in Kenya in 2017 (42). The MomCare program is composed of six elements: standardized care bundle, provider network, digitale exchange platform, health wallet, patient engagement and outcome measurements and provider feedback and improvement.

### Standardized care bundle

**Figure 1:**
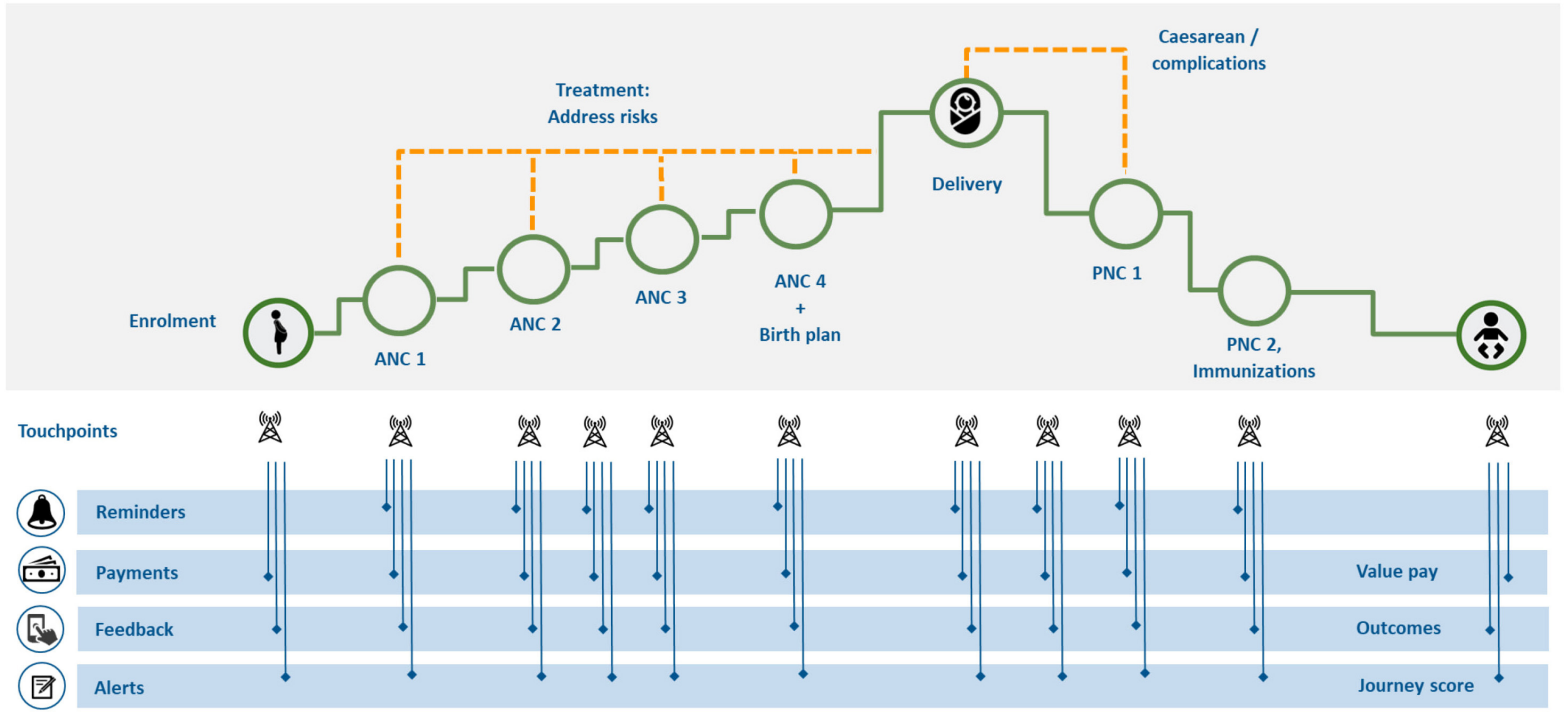
MomCare care pathway.

MomCare follows a predefined and standardized care pathway, which is based on internationally agreed standards for MNCH, quality standards and predetermined costs (43), aligned with the risk profile of the pregnant woman. It covers a bundle of necessary services and interventions such as: antenatal care, essential delivery services and postnatal care. By incentivizing both clinics as well as pregnant women to adhere to the care pathway, MomCare aims to improve MNCH outcomes, including morbidity and mortality. Figure 1 presents the standardized care bundle of MomCare that all participating clinics follow. At various points along the pathway there are interactions between the platform, the enrolled women, and the clinics.

### Provider network

At the start of MomCare, clinics were selected and contracted by PAF to create a network of MNCH providers. The network enables protocol based referrals between clinics. Selection of clinics was based on SafeCare accreditation levels, mobile money readiness and prices. SafeCare is a standard-based care quality improvement methodology (43). The selected clinics were connected to the digital exchange platform, were trained on how to use the platform and received support through SafeCare. PAF started with contracting three clinics in Nairobi and ultimately expanded to 18 clinics across urban and rural areas in 2019. Participating clinics vary from level 2 to 4, covering essential MNCH services.

### Digital exchange platform

MomCare is supported by a digital exchange platform and is compatible with existing information architecture (44). It connects pregnant mothers, healthcare providers and payers (e.g., public health insurers, donors, regional governments) and enables payments, patient engagement, data collection and provides actionable feedback through dashboards. The platform is developed by PAF in the Amazon Web Services and is able to interact with other data sources, such as payment platforms and patient survey tools. The platform adheres to General Data Protection Regulation and local data protection laws to safeguard the privacy of pregnant mothers (44). To connect to the digital exchange platform, each clinic needs to have an internet connection and a desktop computer. As the Kenya National Hospital Insurance Fund (NHIF) uses an online billing process, most clinics are already equipped to work with digital processes.

### Health wallet

As most Kenyans access financial services through their mobile phones, PAF offers enrolled women a “health wallet” on their cell phones to enable payments for health expenses at participating clinics (44). The MomCare “health wallet” operates on the payment platform called M-Tiba, which is developed by CarePay (45). The “health wallet” can be funded by donors and (social) insurance schemes. The “health wallet” is used for two reasons; first it helps the mother establish that she is entitled to care, empowering her and improving both care-seeking behavior and experience. Second, opening the wallet is a digital confirmation that the mother was in the clinic at a specific time for her visit, ensuring that billing can only take place for visits that actually happened. Healthcare providers submit, through M-Tiba, their (claims) data, following the International Statistical Classification of Diseases and Health Related Problems, ICD-10 (46), and receive a bundled payment for a specified set of care activities on a per visit basis. Bonuses are paid based on each woman's adherence to the care pathway (based on a so-called “Journey Score”) and outcome indicators. Data on claims and payments generated by M-Tiba is shared with the MomCare platform for further analysis and creation of performance dashboards.

### Patient engagement and outcome measurements

The platform enables patient engagement throughout the various stages of the care pathway. At enrolment, pregnant women are asked questions on socio-economic status, demographics and obstetric history including pre-existing medical conditions, using a digital form. This allows for the care bundle to be aligned with the pregnant women's risk profile, e.g., a high risk pregnancy will require additional diagnostic and treatment, and maybe also referral for delivery. Along the pregnancy, SMS-based reminders are sent out to increase adherence to the care bundle and feedback is collected first by telephone calls and later by sending out short SMS-based questionnaires. The responses on these questionnaires are processed into patient-reported outcomes by the platform.

### Provider feedback and improvement

**Figure 2:**
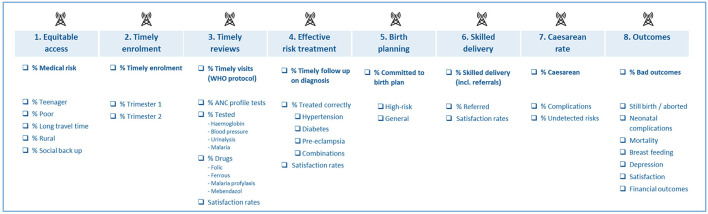
Key performance data collected by the platform.

The platform analyses collected data and provides participating clinics with dashboards showing data on health usage, costs, adherence and outcomes. See Figure 2 for an overview of key performance data collected by the platform. Dashboards are made in PowerBI but only shared as static graphs with the clinics during quarterly data disseminations. To follow each woman in her pregnancy journey, clinics are given access to the patient journey tracker app. This app can be accessed by local staff and login credentials are given and managed by PAF.

The dashboards enable clinics to compare their performance with peers and to identify issues in their service delivery. To support clinics in this process, PAF employs provider support teams lead by a program manager. These teams support clinics in interpreting the data and implementing (quality) improvement programs. Quality improvements are also supported by SafeCare. Each MomCare program manager is able to support about 10–15 health providers.

## VBHC development approach

Since the VBHC approach has rarely been tested and implemented in LMICs, our approach was developed from beginning to end taking local settings and requirements into account. This section describes the three steps of this process: (i) Theory of Change (ToC) development, (ii) VBHC adaptation, and (iii) design of structured feedback loops.

### Theory of change

As a first step, a Theory of Change (ToC) was developed to design the program, and monitor and evaluate the implementation process (47). The ToC describes how MomCare brings about long-term outcomes through a logical sequence of activities, outputs and intermediate outcomes (48). The ToC was developed through several iterative rounds in consultation with stakeholders on different levels, such as government officials of the Kenyan Ministry of Health and members of the Kenyan Obstetrical and Gynecological Society. Interviews and focus group discussions with the involved stakeholders were held to explore the context, challenges, problems, and solutions in providing high quality maternal care. These insights were combined with an extensive document analysis and literature review to design the final ToC version (Appendix 1).

### VBHC adaptation

The VBHC framework consists of six components (49). As a second step of our development approach, the six components of the VBHC framework were adapted to the MNCH context in Kenya as listed in Appendix 2. Adaptation of the VBHC framework is necessary as health systems differ and effects of health system interventions depend on cultural, financial and social context. We specifically focus on outcome measurements and bundled payments as these components were seen as most impactful to patients and providers.

Defining outcomes that reflect the total cycle of care is key within any VBHC initiative. Outcomes should be disease (or in some cases subpopulation-) specific and multidimensional (29). However, designing a valid and reliable outcome set can be complex and time-consuming, especially regarding standardization, which is required to compare between providers and health systems around the world (50). MomCare used an adapted version of the standard set Pregnancy and Childbirth as developed by the International Consortium for Health Outcome Measurements (ICHOM) (51). The ICHOM outcome set includes both clinician-reported outcome measures (CROMs) and patient-reported outcome measures (PROMs) and patient-reported experience measures (PREMs) such as maternal morbidity and birth experience. However, as countries differ in health systems, culture and language, exploring the applicability of outcome sets is required (52). In a previous study in a comparable group of mothers (53), the applicability of the ICHOM set was explored by a two-round feasibility assessment in which pre-selected outcomes were reviewed and finalized by local Kenyan providers and medical experts. In total 14 outcomes were selected as being appropriate in the Kenyan context of which five are patient-reported (53). MomCare used these selected outcomes, which were incrementally implemented along the unfolding of each of the cohorts and perfected over different learning cycles.

An important component of VBHC is reimbursement using bundled payments. Traditionally, providers in Kenya are reimbursed based on fee-for-service of single activities or capitation by the National Hospital Insurance Fund (NHIF) or out-of-pocket payments. A bundled payment is a one-off or periodic lump-sum payment for a range of services delivered by one or more providers based on standardized care pathways with an increasing emphasis on outcomes (54). Unlike fee-for-service, bundled payments transfer financial risk to providers as healthcare providers are expected to provide all necessary care within the bundle. As a result, providers are incentivized to coordinate care across settings, deliver appropriate care and reduce costs over the full care cycle (55). As Kenyan providers are inexperienced using other payment models than fee-for-service and capitation, the program chose an iterative approach by implementing sub-bundles that resemble each phase of the care pathway instead of one bundle that covers the whole pregnancy episode. In total 130 activities, that were separately billed before, were grouped into 32 sub-bundles. The bundled payment model also included a pay-for-performance scheme based on a journey score. The journey score is a standardized risk adjusted metric that quantifies the adherence to the maternity pathway and the care delivered in accordance with the guidelines (10). The score ranges from a minimum of 0 (no care received) to a maximum of 5 (well-attended and managed journey). In order to maintain provider involvement and maximize effects, bonus payments were made available based on the patient journey score and providers received actionable insights (risk stratification, appointment reminders) and clinical insights (data disseminations). MomCare does not apply any penalties when journey scores or outcomes decline.

### Feedback loops

As a third step, we designed a system for structured learning based on data regarding outcomes, outputs and activities. This data, gathered through the platform is analyzed, enriched, and subsequently shared with the clinics through dashboards and clinic visits by PAF fieldworkers. Improvements to the program are implemented every time a new group of mothers (a cohort) was on boarded in the program. In this way, learnings from earlier cohorts can be used to adjust activities to improve outputs and outcomes for later cohorts. In this case study, we describe the roll-out of the MomCare program over seven cohorts, enrolled in the period from 2017 to 2020.

## Uptake and roll-out

In the time-period of this study (2017–2020), MomCare enrolled 8,821 women. In Table 1, the uptake of and roll-out of MomCare is shown over time. Separated by 3 to 4-month intervals (except between cohorts 1 and 2, where there was a longer interval), cohorts of pregnant women were invited to enroll in MomCare at a contracted clinic. The total patient journey takes 45 weeks on average. At the start, admission was set at ≤ 16 weeks of gestation, but this was later widened. At enrolment, information about MomCare was given as well as mobile phone access to the “health wallet.”

**Table 1:** Uptake of and roll-out of MomCare.

	**Cohort**									
	**Urban**							**Rural**		
**Indicator**	**1**	**2**	**3**	**4**	**5a**	**6a**	**7a**	**5b**	**6b**	**7b**
Enrolled Mothers	217	172	517	481	282	901	1,240	824	1,978	2,209
# Hubs	0	1	1	1	1	1	1	-	-	-
# Spokes	2	1	2	3	3	4	6	-	-	-
# Combined hub and spoke	0	0	0	0	0	2	2	4	10	14

Table 1 also shows the number of participating clinics for each cohort. Clinics can have a hub, spoke, or combined hub and spoke status. Clinics having a hub status provide more complex health services when referred to, e.g., in case of a complicated delivery or C-section. Spoke clinics offer basic MNCH care services, including normal skilled deliveries. Clinics with a combined hub and spoke status offer both basic and more complex health services. From cohort 5 onwards, MomCare was expanded to the rural areas of Kakamega and Kisumu (cohorts 5b to 7b).

### Cohort 1

**Figure 3:**
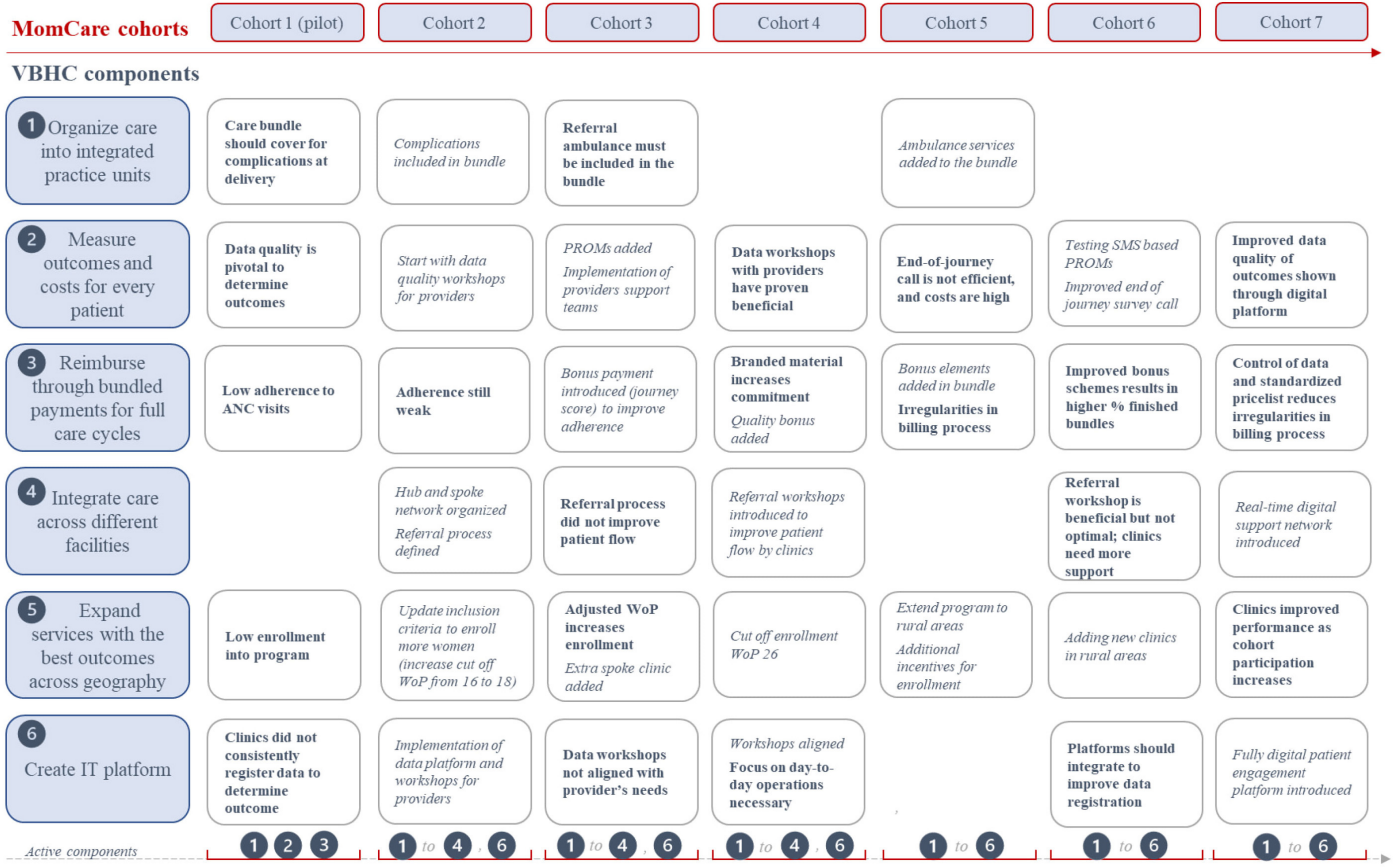
Implementation of VBHC components, learnings and adaptations (learnings are shown in bold, adaptations of components are shown in italic).

The first cohort of MomCare started in Nairobi in November 2017 (Table 1) enrolling 217 women on the digital platform. As shown in Figure 3, three VBHC components were implemented in cohort 1. Two clinics were contracted and connected to the platform to act as an integrated practice unit (IPU) offering a bundle of basic MNCH care services, including four antenatal care (ANC) visits, skilled (normal) delivery service and two PNC visits (including immunizations). Clinics were reimbursed by bundled payments.

### Cohort 2

Cohort 2 started in July 2018, enrolling 172 pregnant women in MomCare. This lower number was caused by a spoke clinic leaving MomCare and limited funding to enroll more women. To address the lack of complications coverage, a hub-and-spoke network (VBHC component 4) was created in cohort 2 to cover for C-sections and basic complications across clinics (see Figure 3). An additional clinic (hub) was connected and together with the spoke clinic a triage and referral process was defined. Secondly, the platform was made suitable to capture and share clinical outcomes and claims data. Finally, the inclusion criteria were widened from 16 to 18 weeks of pregnancy (WoP) to increase the enrolment of pregnant women.

### Cohort 3

In cohort 3, starting in October 2018, a second spoke clinic was added to attract pregnant women and to strengthen the hub and spoke network. Also PROMs were added to the outcomes that were systematically collected from enrolled women and bonus payments based on the journey score were introduced. To help clinics learn and improve PAF support teams were formalized. The cut-off point to enroll in MomCare changed to a maximum of 26 weeks of gestation. With these wider inclusion criteria, 517 women enrolled in MomCare.

### Cohort 4

Cohort 4 started in February 2019. As the referral process in cohort 3 did not result in a better patient flow, the hub and spoke network organized a series of workshops to optimize the referral process and synchronize their cooperation. Secondly, the content of the data workshops was better aligned with the needs of employees of participating clinics. In the data workshop more attention was paid to creating commitment at clinics to use data in their day-to-day operations. Thirdly, investments were put in strengthening the hub and spoke network by adding an extra spoke clinic to shorten travel time for enrolled women. In total 481 pregnant women enrolled in the program.

### Cohort 5

In May 2019, MomCare implemented VBHC component 5 by expanding services to the rural areas of Kakamega and Kisumu. Now, all components of the VBHC framework were implemented. Other changes were the use of standardized pricelists, further improvement of the referral process, providing free maternity goods incentivizing women to enroll into MomCare, and integration of patient-reported outcome data collection tools (end-of-journey calls and SMS-based questionnaires) to simplify data capturing. From cohort 5 onwards, a distinction is made between the women enrolled in the urban area (cohorts “a”) and women enrolled in rural areas (cohorts “b”). In cohort 5b, four clinics were contracted to offer the basic bundle including C-sections and treatment of complications. In total 824 women enrolled in cohort 5b.

### Cohort 6

Cohort 6 started in September 2019. Based on the short-cycle learnings of cohort 5, several improvements were made. First, as transport costs can be a barrier to health care access (56, 57) ambulance services were added to MomCare. Secondly, access to data on outputs and outcomes was made easier and dashboards were improved to be used on a daily basis by clinics and monitoring staff. Thirdly, control of billing processes and provider engagement was tightened to prevent irregularities (such as double or false claims). Finally, the bonus payment was improved by adding adherence elements. In cohort 6, nine additional clinics were contracted, and 2,879 women enrolled in MomCare.

### Cohort 7

In cohort 7, which started in January 2020 and coincided with COVID-19 lockdowns per March 2020, a fully digital patient engagement platform was introduced including digital enrolment, SMS-based reminders and outcome measurements. Second, a digital support network was introduced to help clinics improve their health services. The digital platform also enabled a patient journey tracker app, which can be used by clinics to follow each patient in their pregnancy journey. Six additional clinics were contracted and 3,449 women enrolled into the program, making this the largest cohort in the program. In cohort 7, SMS-based survey questions on mental health were introduced.

### Progress over time

Over time, more clinics were added, more components of the VBHC framework were implemented, and more pregnant women were enrolled. Progress of the program was tracked using various indicators. The overall impact of the program is reflected in the reach (number of enrolees), the Journey Score, and the variety of outcome measures tracked per enrolee. The reach of the program increased more than 15-fold, from 217 enrolees in cohort 1 to 3,449 enrolees in cohort 7a and 7b combined.

**Figure 4:**
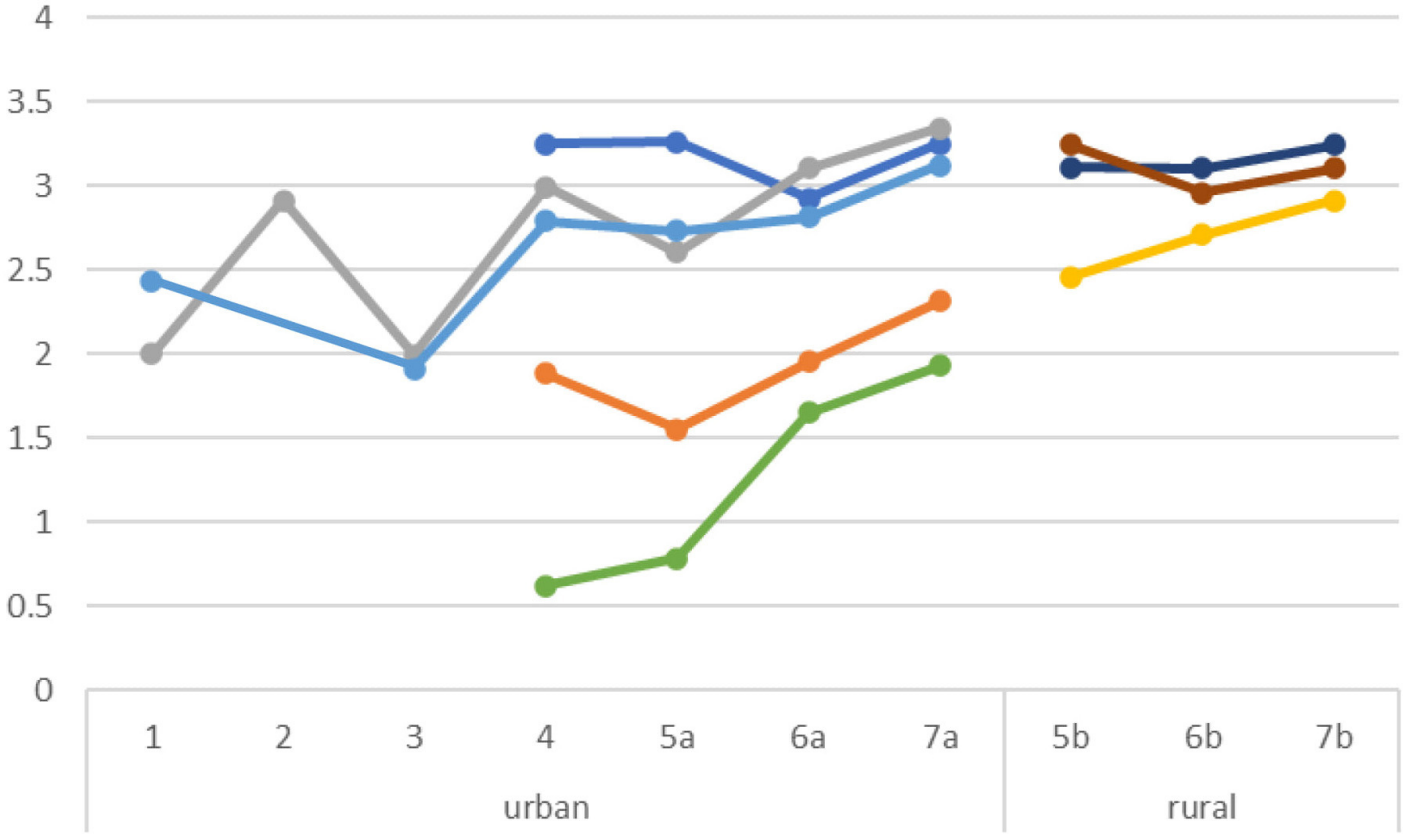
Journey Score per clinic. Each color represents a different clinic. Scores on a scale of 0–5.

The Journey Score is a measure of adherence to the care pathway. Figure 4 shows the patient journey scores of individual clinics that participated in three or more cohorts. This data shows the effects of the short and long cycle learnings for each clinic. Clinics with lower baseline scores are clearly improving, and over time the differences between clinics become smaller. A less strong improvement is shown for the clinics operating in rural areas, but their baseline scores were already high.

## Discussion

The objective of MomCare was to improve outcomes of MNCH care in LMICs through access to high quality care based on the VBHC framework. In HICs, volume-driven transitions aimed at high quality care have shown that unwanted cost increases can occur (54), something especially health systems in LMICs cannot afford. Therefore, in LMICs, value-driven health systems could be a game changer, as they are characterized by generating maximum value for patients by cost-effectively achieving the best possible outcomes. A volume-driven focus on access alone threatens health equity and the roll-out of universal health coverage (56, 58). This paper assesses the determinants of transitioning in LMICs from a volume-driven system toward a value-driven system, as per the VBHC framework (1, 9).

The VBHC literature does not provide an implementation methodology and experiences with VBHC framework implementation from HICs cannot be simply copied to the LMIC context. In LMICs, like Kenya, the healthcare landscape is fragmented, with a variety of funders (e.g., government, social insurance, donors) and relatively large differences in healthcare access between regions. This prompted MomCare to take an incremental cohort-based implementation approach of the VBHC framework, building on a scalable platform, and introducing VBHC components gradually. An advantage of the incremental cohort-based approach is that it increases accessibility of care across geographies, while simultaneously improving quality of care, in a relatively short time. This approach allows for co-creation, involving local stakeholders and it enables that services are continuously adapted to the health seeking behavior of the customers. Gradually, a VBHC ecosystem around MNCH was created and continuously improved: a referral system was implemented within a hub-and-spoke network of providers, a system was built to measure clinician and patient-reported outcome measurements, a digital platform was created to enable payments, data capturing and benchmarking, and bundled payments and outcome-based bonus payments were introduced.

In this cohort-based implementation approach, data-driven learning for PAF as well as for the healthcare providers took place at two different speeds: through short learning cycles vs. long learning cycles. Short learning cycles refer to improvements implemented from one cohort to the next and were typically related to care utilization and adherence to care. An example of a short learning cycle is that the model started with a very basic care pathway, but it was soon realized that there were women who needed care for more complicated pregnancies, hence the approach to expand the care pathway to include complicated services and include clinics in the provider network that provide those services. Long learning cycles refer to improvements based on data that is available only at the end of the full care cycle continuum that takes into account the antenatal, delivery and post-natal period of pregnancy estimated at a total of 45 weeks. Before the full care cycle of a cohort (cohort t) is completed, one or two new cohorts (cohorts t+1, t+2) have already started. While short learning cycles bring improvements from cohort t to cohort t+1, long learning cycles bring improvements from cohort t to cohort t+3. An example of long cycle learning is the additional training offered to providers around breastfeeding: train providers to inform women about the importance of breastfeeding and how to deal with challenges experienced by mothers. This improvement was based on outcomes that could only be measured at the end of a full care cycle. A prerequisite is that the implementing team and the providers must have a strong learning culture (59) that allows for course correction whenever necessary and is aided by the data driven approach that PAF takes.

During roll-out we identified several challenges, three of which still require further fine tuning today. First, it remains paramount to improve quality of data registries and consistency of data capturing by providers, especially data that is not captured automatically through billing processes such as mortality rates. Additional training of providers proved key to improve data collection and usage (60). Increased transparency of provider performance, benchmarking with other (competing) providers and financial rewarding through bonuses are all factors that impact the willingness of providers to collect and share data. Continued effort is needed to motivate and enable providers to reliably capture and report process and outcome data. Second, as the providers are used to being paid on a fee-for-service basis or *via* capitation, shifting financial risk from payer to provider by introducing full bundled payments was experienced as a bridge too far. It proved impossible (as yet) to shift financial risks to providers completely by introducing one bundled payment that covers the total patient journey. Therefore, the program grouped billable services into smaller bundles. Further investment is needed to train providers on how to manage financial risks related to bundled payments. A third challenge concerns the relatively limited validity of Western patient-reported outcome measures. Outcome measures need to be adapted to what matters to pregnant women with different cultural backgrounds living in Kenyan urban and rural areas. Further research is needed to validate such adapted questionnaires to provide reliable PROMs for women's views of high quality MNCH care.

The success of implementing a VBHC based digital platform in LMIC settings, such as Kenya, depends on several enabling factors. These include having an IT infrastructure in place to enable digital communication and data collection, a high uptake of mobile phones in the community, the availability of mobile money, and management buy-in at clinic level to learn and to improve care. More specific enablers are the deployment of a provider support team to train and support clinics with VBHC interventions and digital skills (61). Another enabling factor relates to the position of a trusted third party like PAF to connect payers to providers and to create a high-trust environment in which clinics are willing to participate in a program.

Finally, the scalability of the program is key. The impact of MomCare on MNCH in Kenya depends on the ability to scale up the technology and bring the VBHC approach to MNCH to more women in more regions, both urban and rural. Various key elements of the MomCare program are easy to scale up. The technology is suitable for processing many new enrolments within a short period of time. The development of the digital platform requires a high investment and an incremental approach, but operational costs will decrease as more women enroll into MomCare. Since the start of MomCare, operational costs of the platform decreased from an estimated 4 USD for each enrolled woman to an estimated 1 USD. The supporting analysis tools of the digital platform, such as the dashboard and benchmarking tools, are easy to scale up. This also applies to outcome measurements along the care pathway. The data collection of both clinical and patient-reported outcomes is fully integrated into the digital platform.

Elements that are considered a greater challenge to scaling up MomCare are related to its provider network and the interactions with contracted clinics. MomCare aims to improve care processes and is highly data driven, but most staff members of clinics are not sufficiently trained in working with data and improving care processes. This requires intensive training and support by the MomCare provider support teams. But scaling up provider support teams is challenging because it requires specific knowledge about quality improvement processes, relationship management, data analytics and operational processes. We have learnt that significant support is needed for providers in the first 6–9 months of the program, to ensure an effective shift in mindset toward data driven care. Finally, as MomCare uses standardized care pathways and financially depends on payers such as donors and NHIF, it appears that MomCare is sensitive to unexpected system shocks, such as political instability, economic setbacks and unforeseen events, such as COVID-19. Adapting MomCare to these system shocks is challenging. Especially when it requires adaptations of the fundamentals such as the digital platform or care pathway.

Elements of MomCare that are difficult to scale up mainly relate to the acceptance of new payment models by clinics. Currently most MNCH clinics in Kenya are paid through fee-for-service (FFS), through out-of-pocket (OOP) or through the National Hospital Insurance Fund (NHIF). Implementing alternative payment models such as bundled payments is difficult. Clinics have difficulty accepting alternative payment models, because financial incentives are not well-understood, and are seen as inappropriate. This is especially true for alternative payment models allocating more financial risks to providers. Scaling up is also difficult because a variety of payers is involved. Each payer has its own objectives and requirements for reporting and assessment, which increases transaction costs. Ideally funds are pooled to cover the operational expenses and health care costs of MomCare. But all-in-all, by using existing platforms for mobile money and SMS communications, the program managed to increase the sophistication of services provided while keeping transaction costs low (61).

## Conclusion

This case study shows that implementation of the VBHC framework in an LMIC setting is possible with some adaptations to the local context. The digital platform with integrated mobile money and SMS-based communications was key to the success of the program. Participating clinics showed progress in improving MNCH outcomes. The first results are positive, but more research is needed to provide more clarity on its impact on costs and quality. The cohort-based approach created short cycle and long cycle feedback loops enabling gradual implementation of the six VBHC components in co-creation with stakeholders. We recommend that this cohort-based implementation approach as well as the integrated digital platform design as described in this case report is used for other conditions and patient groups, such as ischaemic heart diseases and diabetes mellitus type 2, in order to incrementally build and adapt a customized VBHC strategic framework for addressing multiple diseases and conditions with high disease burden in LMICs.

## Data availability statement

The data analyzed in this study is subject to the following licenses/restrictions: The data that support the findings of this study are available from PharmAccess Foundation, but restrictions apply to the availability of these data, and thus are not publicly available. Data are available from the corresponding author upon reasonable request and with permission of PharmAccess Foundation. Requests to access these datasets should be directed to dohmen@eshpm.eur.nl.

## Author contributions

PD, EW, and TD contributed to conception and design of the case report, collected, and organized the data for the case study. PD wrote the first draft of the manuscript. PD and EV finalized the manuscript. All authors contributed to manuscript revision, read, and approved the submitted version.

## Conflict of interest

The authors declare that the research was conducted in the absence of any commercial or financial relationships that could be construed as a potential conflict of interest.

## Publisher's note

All claims expressed in this article are solely those of the authors and do not necessarily represent those of their affiliated organizations, or those of the publisher, the editors and the reviewers. Any product that may be evaluated in this article, or claim that may be made by its manufacturer, is not guaranteed or endorsed by the publisher.
