# Supplementary material for: Implementing value-based healthcare using a digital health exchange platform to improve pregnancy and childbirth outcomes in urban and rural Kenya
Source: Front Public Health. 2022 Nov 17;10:1040094. doi: 10.3389/fpubh.2022.1040094 (PMC9712749; doi:10.3389/fpubh.2022.1040094)
Supplement: Supplementary file 1 [file Table_1.DOCX]

**Appendix 1:** Theory of Change^^[[1]](#footnote-1)^^

**
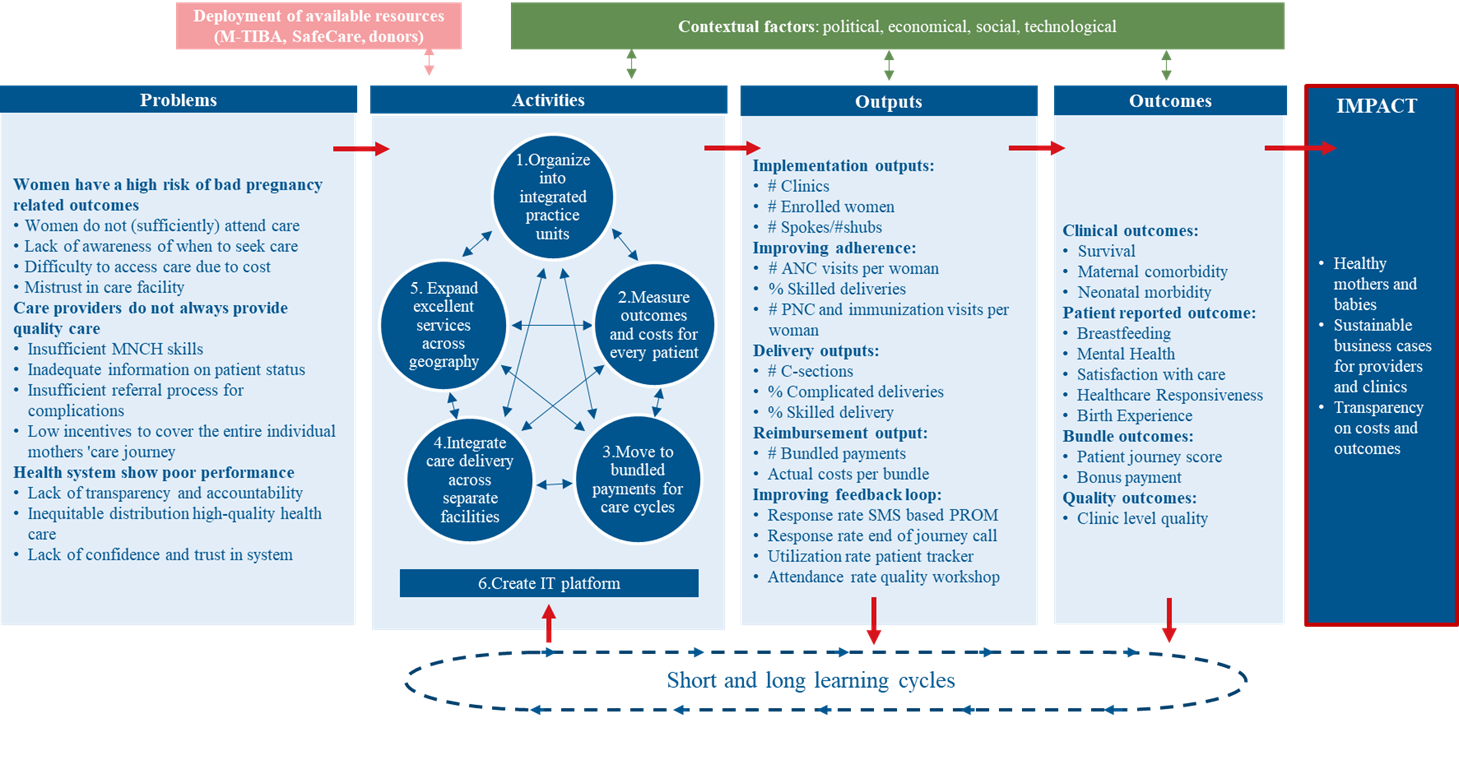
**

1. In an adapted form, this figure also appeared in [44] [↑](#footnote-ref-1)
