# Supplementary material for: Implementing value-based healthcare using a digital health exchange platform to improve pregnancy and childbirth outcomes in urban and rural Kenya
Source: Front Public Health. 2022 Nov 17;10:1040094. doi: 10.3389/fpubh.2022.1040094 (PMC9712749; doi:10.3389/fpubh.2022.1040094)
Supplement: Supplementary file 2 [file Table_2.DOCX]

**Appendix 2:** How each component of the VBHC framework was translated into context-specific activities.

| **1** | **Organize into integrated practice unit (IPU)** | **2** | **Measure outcomes and costs for every patient** | **3** | **Move to bundled payments for care cycles** | **4** | **Integrate care delivery across separate facilities** | **5** | **Expand excellent services across geography** | **6** | **Create IT platform** |
| --- | --- | --- | --- | --- | --- | --- | --- | --- | --- | --- | --- |
| a | Redefining of care pathways | a | Collecting patient reported (PROMs) and clinician reported outcomes (CROMs) | a | Implementation of bundled payments | a | Introducing hub and spoke model | a | Use a cohort-based approach (cohorts 1-7) | a | Implementation of digital payment platform (enabling data collection of CROM) |
| b | Implementation of quality workshops and quality certification (SafeCare) | b | Implementation of outcome measurements by digital tools and use of billing data | b | Offering comprehensive coverage MNCH | b | Implementation of referral system (content package across different facilities) | b | Start of program in urban area (Nairobi) and expansion to rural areas (Kakamega and Kisumu) | b | Implementation of patient journey tracker app (enabling data collection of PROM) |
| c | Investments in medical equipment and quality improvements | c | Implementation of journey score | c | Implementation of outcome-based bonus payments |  |  |  |  | c | Implementation provider of performance dashboard |
|  |  |  |  | d | Implementation of contracting and monitoring process between PAF and clinics |  |  |  |  |  |  |
